# A novel site on dual-specificity phosphatase MKP7/DUSP16 is required for catalysis and MAPK binding

**DOI:** 10.1016/j.jbc.2022.102617

**Published:** 2022-10-19

**Authors:** Shanelle Shillingford, Lei Zhang, Yulia Surovtseva, Sam Dorry, Elias Lolis, Anton M. Bennett

**Affiliations:** 1 Department of Pharmacology, Yale University School of Medicine, New Haven, Connecticut, USA; 2 Department of Chemistry, Yale University, New Haven, Connecticut, USA; 3 Yale Center for Molecular Discovery, Yale West Campus, West Haven, Connecticut, USA; 4 Yale Center for Molecular and Systems Metabolism, Yale University School of Medicine, New Haven, Connecticut, USA

**Keywords:** MAPK, protein phosphatases, allosteric site, phosphorylation, signal transduction, DUSP, dual-specificity phosphatase, ERK1/2, extracellular signal-regulated kinases 1 and 2, FBS, fetal bovine serum, JNK, Jun NH2 terminal kinase, KIM, kinase-interacting motif, MAPK, mitogen-activated protein kinase, MKP, MAPK phosphatases, pNPP, para-nitrophenyl phosphate, PTP, protein tyrosine phosphatase, SRE, serum-responsive element

## Abstract

The dual-specificity phosphatases responsible for the inactivation of the mitogen-activated protein kinases (MAPKs) are designated as the MAPK phosphatases (MKPs). We demonstrated previously that MKP5 is regulated through a novel allosteric site suggesting additional regulatory mechanisms of catalysis exist amongst the MKPs. Here, we sought to determine whether the equivalent site within the phosphatase domain of a highly similar MKP family member, MKP7, is also important for phosphatase function. We found that mutation of tyrosine 271 (Y271) in MKP7, which represents the comparable Y435 within the MKP5 allosteric pocket, inhibited MKP7 catalytic activity. Consistent with this, when MKP7 Y271 mutants were overexpressed in cells, the substrates of MKP7, p38 MAPK or JNK, failed to undergo dephosphorylation. The binding efficiency of MKP7 to p38 MAPK and JNK1/2 was also reduced when MKP7 Y271 is mutated. Consistent with reduced MAPK binding, we observed a greater accumulation of nuclear p38 MAPK and JNK when the MKP7 Y271 mutants are expressed in cells as compared with WT MKP7, which sequesters p38 MAPK/JNK in the cytoplasm. Therefore, we propose that Y271 is critical for effective MAPK dephosphorylation through a mechanism whereby binding to this residue precedes engagement of the catalytic site and upon overexpression, MKP7 allosteric site mutants potentiate MAPK signaling. These results provide insight into the regulatory mechanisms of MKP7 catalysis and interactions with the MAPKs. Furthermore, these data support the generality of the MKP allosteric site and provide a basis for small molecule targeting of MKP7.

The mitogen-activated protein kinase (MAPK) phosphatases (MKPs) are a group of ten dual-specificity phosphatases (DUSPs) that dephosphorylate and inactivate the MAPKs. The MAPKs are grouped as the growth factor–responsive extracellular signal-regulated kinases 1 and 2 (ERK1/2), the stress-responsive c-Jun NH_2_ terminal kinases 1 and 2 (JNK), and p38 MAPK at their threonine and tyrosine active site residues (1, 2). The MAPK’s play very important roles in a variety of cellular functions, and their aberrant signaling has been linked to several diseases including cancer and diabetes (3, 4). As such, the MAPKs are actively studied as therapeutic targets, with several small molecule inhibitors already successfully developed (5, 6, 7, 8). Due to the involvement of the MAPKs in a wide range of signaling pathways (9), targeting downstream substrates and upstream regulators of the MAPKs as additional and/or complementary therapeutic targets is important. However, development of modulators against the MKPs as potential therapeutic targets has proven challenging, largely due to the high sequence similarity amongst the DUSP family and high structural conservation surrounding the active site catalytic cysteine (10).

The MKPs have a conserved MAPK-docking site at the N-terminus designated as the kinase-interacting motif (KIM) which is essential for both substrate recognition and orientation (11, 12), in addition to a well-conserved catalytic pocket in the C-terminus comprising the motif, HC(X)_5_RS (13). The active site cysteine resides in the P-loop and performs a nucleophilic attack on the incoming phosphate group of the substrate, while a thiolate intermediate leads to a conformational change in the active site pocket that encloses the substrate (14). Simultaneously, an aspartic acid that is resident in what is designated as the WPD loop or the D-loop protonates the substrate allowing for substrate release while a neighboring arginine also in the P-loop stabilizes the phosphate intermediate (15). This first step of catalysis is followed by hydrolysis of the phosphate group by a water molecule leading to the release of an inorganic phosphate ion and enzyme (15, 16, 17).

The active site cysteine has a low pKa that causes this residue to be negatively charged in the MKPs and in high throughput screens often leading to the identification of small molecule inhibitors that have reactivity to the cysteine thiol group and/or charged molecules with extremely poor membrane permeability (18). The difficulty in generating small molecule inhibitors with desirable drug properties and specificity to a particular MKP has deemed the MKPs, like members of the broader protein tyrosine phosphatase (PTP) family, as largely “undruggable” (18). Progress has been made on this front with the discovery of a low potency, allosteric inhibitor of MKP5 that circumvents the issues of active site inhibitors (19). Since the MKPs share high sequence similarity in this region, this suggests a new regulatory site that may be conserved amongst the MKPs.

To investigate whether the comparable region of MKP5 conveys similar properties in other MKPs, we investigated the MKP with the highest similarity to that of MKP5, namely MKP7. MKP7 is a 665 aa DUSP, encoded by the *DUSP16* gene, which maps to human chromosome *12p12* and exhibits 44% sequence similarity within the PTP domain as compared with MKP5 (20, 21). In addition to the KIM and catalytic core conserved in all MKPs (13), MKP7 has an extended C-terminal region containing both a nuclear export signal and a nuclear localization signal. When overexpressed in mammalian cells, MKP7 is localized predominantly to the cytoplasm (20). MKP7 preferentially inactivates JNK1/2, followed by p38α/β MAPK (JNK1/2 >>p38 MAPK>ERK) and is capable of binding to all three of the MAPKs (21, 22). Initially, two established docking sites for both p38α/β MAPK and JNK on MKP7 were identified, one in the N-terminus kinase-binding domain and one in the catalytic domain (22). However, it was revealed that an additional docking domain exists with an FXF motif (also known as the F-site) that is required for MKP7 recognition and JNK inactivation (23). We noted that the primary sequence containing the allosteric site on MKP5 is conserved in MKP7, suggesting that this region might not only be involved in MAPK binding and recognition but also MKP7 catalysis. Here, we sought to establish the relevance of this region for MKP7 activity and substrate interactions.

We demonstrate that like MKP5, MKP7 contains a similar region defined by Y271 that is required for MKP7 catalysis. Y271 in MKP7 was found to be involved in p38 MAPK and JNK binding and subcellular localization of these MAPKs. Collectively, this study has revealed a mode of MAPK interaction and regulation of MKP7 catalysis and further suggests that the MKP5 allosteric pocket is a common regulatory property amongst the MKPs.

## Results

### MKP7 shares sequence and structural similarities with the allosteric pocket of MKP5

**Figure 1:**
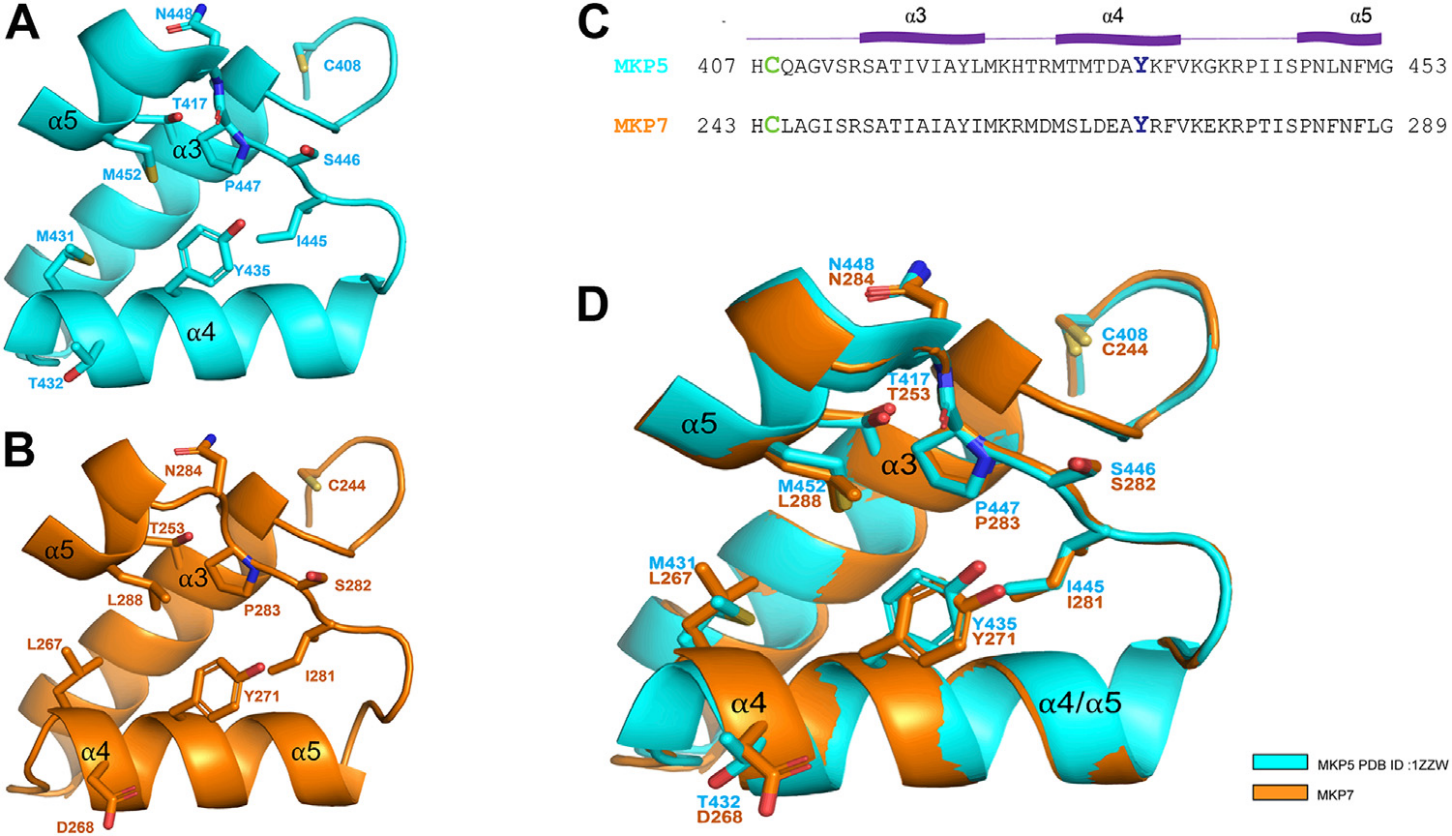
**Structural alignment of allosteric pocket in MKP5 and MKP7.** The *apo*-crystal structure of the PTP domain of MKP5 WT available *via* RCSC Protein Data Bank was compared with a model of the PTP domain of MKP7 WT generated by the software AlphaFold (25, 26). *A*, shows the allosteric pocket of MKP5 with the key Y435 residue that is required for MKP5 catalytic activity (19). *B*, a model of the putative allosteric pocket in MKP7, highlighting the equivalent residue in MKP7, namely Y271. *C*, sequence alignment of the residues encompassing the catalytic pocket and allosteric pocket in both MKP5 and MKP7. The conserved active site cysteine is highlighted in *green* and the conserved tyrosine in the allosteric site is highlighted in *blue*. *D*, structural alignment of MKP5 and MKP7 were done using the PyMol software to determine conformational similarities. MKP, MAPK phosphatase; PTP, protein tyrosine phosphatase.

We have previously shown that the allosteric site identified in the catalytic domain of MKP5 is formed by α helices, α3, α4, and α5, in addition to the α4–α5 loop (Fig. 1*A*) (19). This allosteric pocket in MKP5 is located approximately 8 Å from the catalytic sulfhydryl of Cys 408 (C408) and contains a critical residue (Y435) that stabilizes binding between MKP5 and the small molecule inhibitor that binds this region through a π-π stacking interaction (19). Further support for the conclusion that an allosteric site is associated with the catalytic activity are mutations of Y435 and other residues (M431 and T432) comprising this site that result in a dramatically impaired MKP5 binding of the allosteric inhibitor and loss of MKP5 catalytic activity (19).

Inspection of the MKP5 allosteric region to other MKPs demonstrates that this site is similar, and Y435 is highly conserved amongst the MKP family. Although the MKP5 allosteric inhibitor has reduced efficacy on MKP7 catalytic activity, we focused on whether Y271 of MKP7 (corresponding to MKP5 Y435) is important for catalytic activity. To gain insight into the structural similarity between the allosteric pocket of MKP5 and the putative allosteric site of MKP7, we compared the crystal structure of this region (helices α3, α4, α5, and the α4–α5 loop) of the catalytic domain of the *apo*-crystal structure of MKP5 (1ZZW) (24) with the modeled structure of *apo*-MKP7 using the AlphaFold protein structure database (25, 26) (Fig. 1*B*). The entire region constituting the allosteric site (Fig. 1*C*), including other key residues that impact MKP5 catalytic activity, are well-aligned structurally with MKP7 (Fig. 1*D*). Specifically, Y271 of MKP7 is overlayed with Y435 in MKP5 and is similarly distant from the catalytic cysteine (C244). The observation that there is a high degree of structural similarity within this region involved in forming an allosteric site for MKP5 raises the possibility that Y271 represents a critical residue involved in MKP7 catalysis.

### Y271 is required for MKP7 catalytic activity

**Figure 2:**
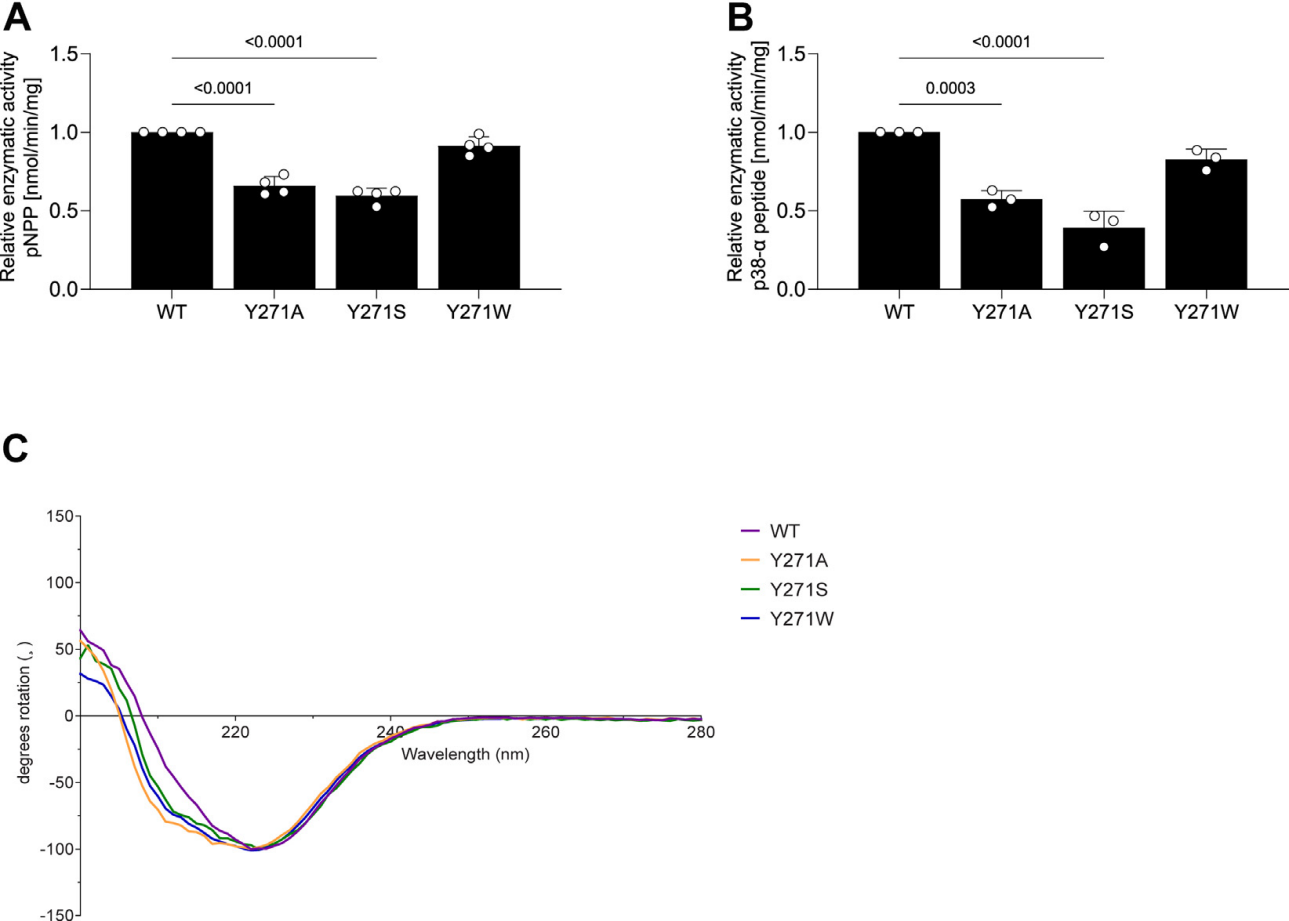
**Effect of Y271 on MKP7 catalytic activity.** The catalytic domain of His-tagged MKP7 WT, Y271A, Y271S, Y271W were purified from *Escherichia coli*. MKP7 catalytic activity was determined in *A*, using the small molecule substrate, *para*-nitrophenyl phosphate and *B*, using p38α MAPK phospho-mimetic peptide substrate. Data represent 3 to 4 independent experiments and the indicated statistical values were generated using one-way ANOVA test. *C*, circular dichroism (CD) was used to evaluate the folding of all MKP7 variants and confirmed that all mutants were appropriately folded. CD spectra were collected in three independent data sets for each mutant and normalized internally. MAPK, mitogen-activated protein kinase; MKP, MAPK phosphatase.

To determine the effects of Y271 on MKP7 activity, we purified the catalytic domain of WT MKP7, along with proteins in this domain containing mutations Y271A, Y271S and Y271W. Using para-nitrophenyl phosphate (*p*NPP) as substrate, we found that Y271A, Y271S, and Y271W retained 66%, 60%, and 91% of their activity relative to that of WT, respectively (Fig. 2*A*). To investigate the effect of Y271 on the catalytic activity with a physiological MKP7 substrate, an 11 amino acid phospho-peptide of p38α MAPK with the regulatory threonine and tyrosine residues phosphorylated was used in a malachite green assay. Upon incubation of the p38α MAPK phospho-peptide with WT and mutant MKP7 proteins, we found that the mutant proteins Y271A, Y271S, and Y271W retained 57%, 39%, and 83% of their activity relative to that of WT, respectively (Fig. 2*B*). The differences in the activity of Y271 mutants are likely attributed to loss of intrinsic MKP7 activity as both WT and mutant proteins demonstrated similar protein folding properties (Fig. 2*C*). Thus, either using a small molecule phosphotyrosine mimetic (Fig. 2*A*) or the p38α MAPK phospho-peptide (Fig. 2*B*) as substrates demonstrated a requirement for Y271 for optimal catalysis. Furthermore, the Y271W mutant retained most of its activity relative to that of WT MKP7 likely due to the maintenance of aromaticity of this residue to that of tyrosine in WT MKP7. Taken together, these data support the interpretation that Y271 of MKP7 plays an essential role in regulating catalysis.

### Effect of MKP7 Y271 on MAPK signaling

**Figure 3:**
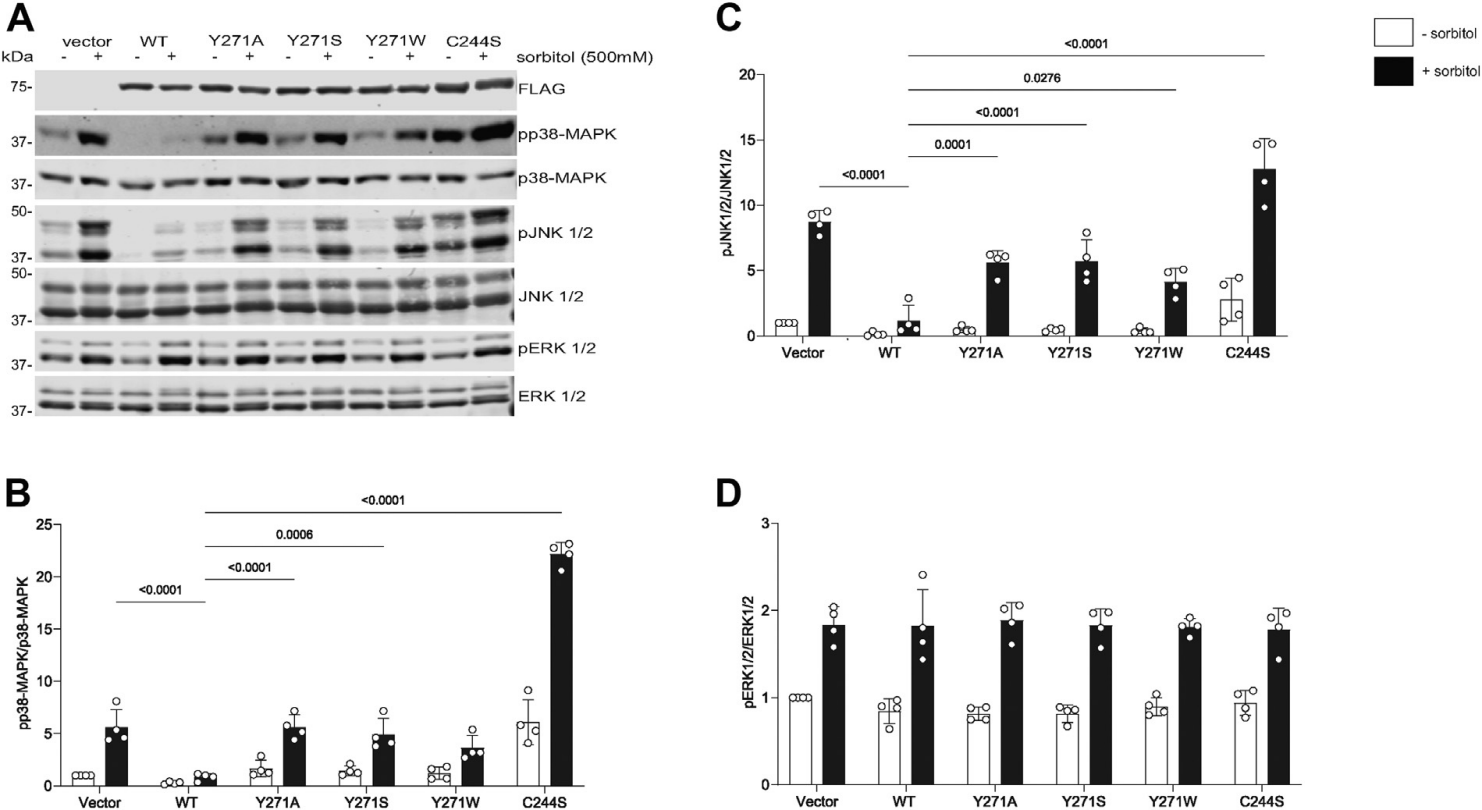
**Effect of MKP7 Y271 on MAPK activation.** Full-length FLAG-tagged MKP7 WT, Y271 mutants, and the catalytically inactive C244S were transiently transfected into COS-7 cells. Cells were placed in starvation media (0.1% FBS) for 24 h followed by 30 min stimulation with 500 mM sorbitol in 0.1% FBS. Cell lysates were prepared, resolved by SDS-PAGE, and immunoblotted with *A*, anti-FLAG, anti-phospho-p38 MAPK, anti-p38 MAPK, anti-phospho-JNK1/2, anti-JNK1/2, anti-phospho-ERK1/2, and anti-ERK1/2 antibodies. Quantitation of immunoblots for *B*, phospho-p38 MAPK/p38 MAPK, *C*, phospho-JNK1/2/JNK1/2 and *D*, phospho-ERK1/2/ERK1/2 was performed by Li-COR and graphs represent the mean ± SEM of 3 to 4 independent experiments. Statistical significance shown was generated using a two-way ANOVA. ERK, extracellular signal-regulated kinase; MAPK, mitogen-activated protein kinase; MKP, MAPK phosphatase.

Given the identified contribution of Y271 toward MKP7 catalytic activity, we next investigated the effect of this residue on full-length MKP7 in a cellular context. FLAG-tagged full-length MKP7 WT, Y271A, Y271S, Y271W, and the catalytically inactive mutant C244S (20) were each transiently transfected into COS-7 cells followed by stimulation with 500 mM sorbitol to activate the stress-responsive MAPKs. When cells were stimulated with sorbitol, phosphorylation of p38 MAPK and JNK1/2 was induced by ∼5- and 9-fold, respectively, as compared with unstimulated cells (Fig. 3*A*). As expected, overexpression of WT MKP7 resulted in the dephosphorylation of both p38 MAPK and JNK1/2 following sorbitol-induced activation as compared with vector control transfectants (Fig. 3, *A*–*C*). Consistent with the lack of substrate selectivity of ERK1/2, overexpression of WT MKP7 did not affect sorbitol-induced ERK1/2 phosphorylation levels (Fig. 3, *A* and *D*). In contrast, overexpression of MKP7 Y271A, Y271S, and Y271W resulted in 7-, 6-, and 4-fold increase respectively, in p38 MAPK phosphorylation as compared with WT in sorbitol-treated cells (Fig. 3, *A* and *B*). Similarly, sorbitol-treated cells exhibited a 6-fold increase in JNK1/2 phosphorylation when Y271A and Y271S were overexpressed and a 4-fold increase when Y271W was overexpressed as compared with WT (Fig. 3, *A* and *C*). When compared to the catalytically inactive and substrate-trapping MKP7 C244S mutant, the MKP7 Y271 mutants were not as effective at inducing either p38α MAPK or JNK1/2 hyperphosphorylation as compared with WT. These results suggest that unlike MKP7 C244S, the MKP7 Y271 mutants do not efficiently trap and subsequently accumulate phosphorylated MAPKs in a dead-end complex. Together, these data demonstrate that Y271 is critical for MKP7 catalytic activity in a cellular context and functions as a dominant-interfering mutant.

### Contribution of MKP7 Y271 for p38 MAPK and JNK interactions

**Figure 4:**
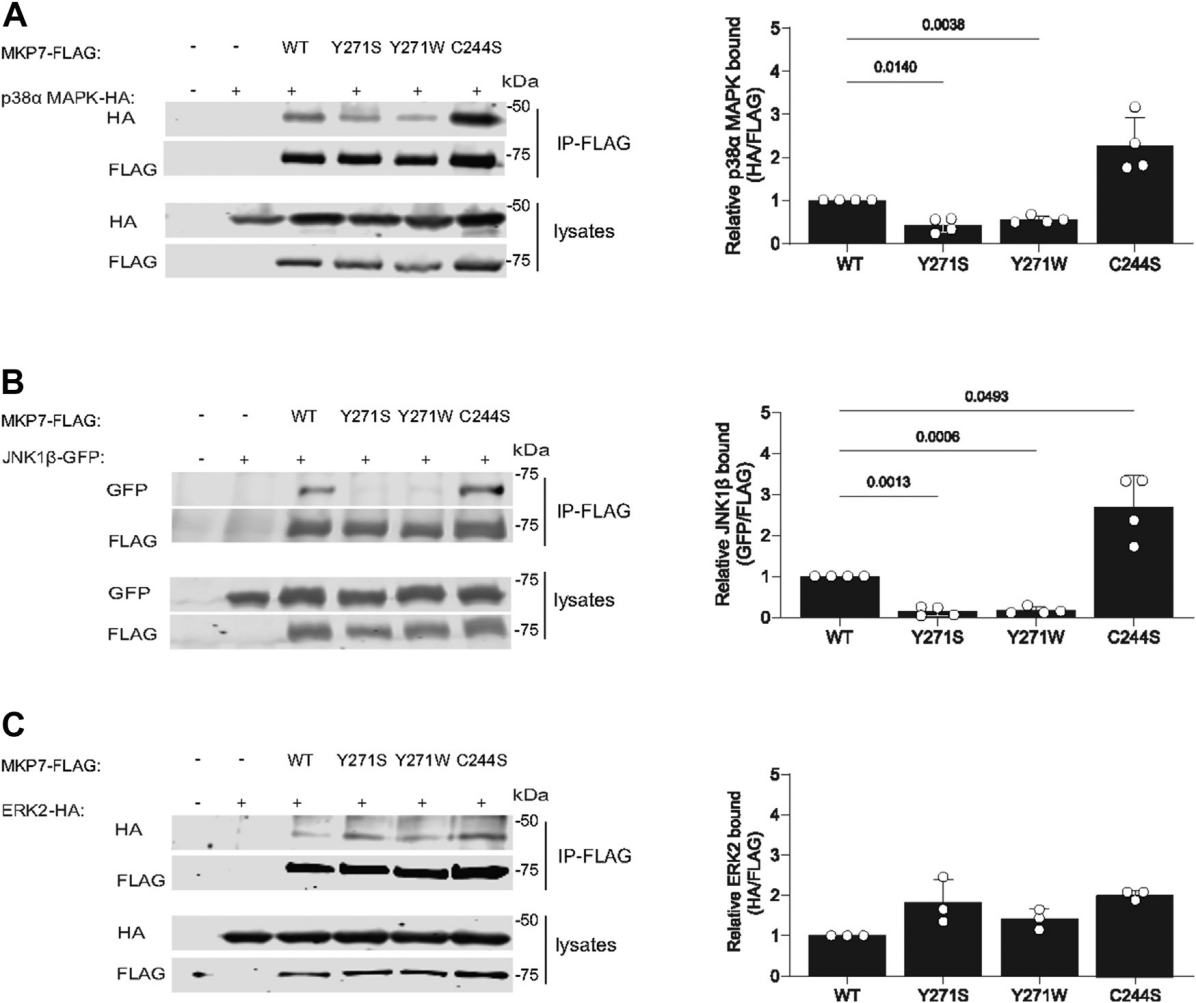
**Effect of MKP7 Y271 on MAPK binding interactions.** Full-length FLAG-tagged MKP7 WT, Y271 mutants, and C244S were transiently transfected with p38α MAPK-HA, JNK1-β-GFP, or ERK2-HA into COS-7 cells for 48 h. Cells were harvested and lysed, followed by immunoprecipitation with anti-FLAG antibodies. *A*, anti-FLAG immune complexes and whole cell lysates were immunoblotted with anti-HA and anti-FLAG antibodies. *B*, anti-FLAG immune complexes and whole cell lysates were immunoblotted with anti-GFP and anti-FLAG antibodies. *C*, anti-FLAG immune complexes and whole cell lysates were immunoblotted with anti-HA and anti-FLAG antibodies. Graphs shown to the right represent quantitation of *A*, p38α MAPK-HA/FLAG-MKP7, *B*, JNK1-GFP/FLAG-MKP7 and *C*, ERK2-HA/FLAG-MKP7. Data represent the mean ± SEM of three to four independent experiments. Statistical significance shown was generated using a two-way ANOVA. ERK, extracellular signal-regulated kinase; MAPK, mitogen-activated protein kinase; MKP, MAPK phosphatase.

It has been shown that MKP7 interacts with JNK through the FXF motif (23). Furthermore, our modeling analysis between the MKP5 allosteric pocket in which Y435 is contained and JNK suggested that this region in MKP5 might also serve as a JNK docking site (19). Therefore, we tested the ability of Y271 on MKP7 to mediate complex formation with the MAPKs. FLAG-tagged MKP7 WT, Y271S, and Y271W (Y271A mutant was not used because Y271S has an equivalent effect on MKP7 catalytic activity) along with C244S were cotransfected into COS-7 cells with either p38α MAPK-HA, JNK1-β-GFP, or ERK2-HA followed by immunoprecipitation of FLAG-tagged MKP7 variants. We found that MKP7 WT complexed with p38α MAPK, however, Y271S and Y271W mutants exhibited 50% reduced p38α MAPK complex formation compared with WT MKP7 (Fig. 4*A*). Consistent with the substrate-trapping properties of the MKP7, C244S mutant p38α MAPK bound up to 3-fold higher levels, as compared with WT (Fig. 4*A*). Remarkably, binding to JNK1β was completely abrogated in MKP7 Y271S and Y271W as compared with WT MKP7, whereas the C244S mutant bound ∼3-fold higher levels of JNK as compared with WT (Fig. 4*B*). As expected, the C244S mutant bound increased levels of ERK2, but no differences in ERK2 binding were observed with the MKP7 Y271 mutants (Fig. 4*C*). These results demonstrate that Y271 in addition to contributing to the catalytic activity of MKP7 is also involved in mediating complex formation with its physiological substrates, p38α MAPK and JNK.

### MAPK binding to MKP7 Y271 is a prerequisite for MKP7 catalysis

**Figure 5:**
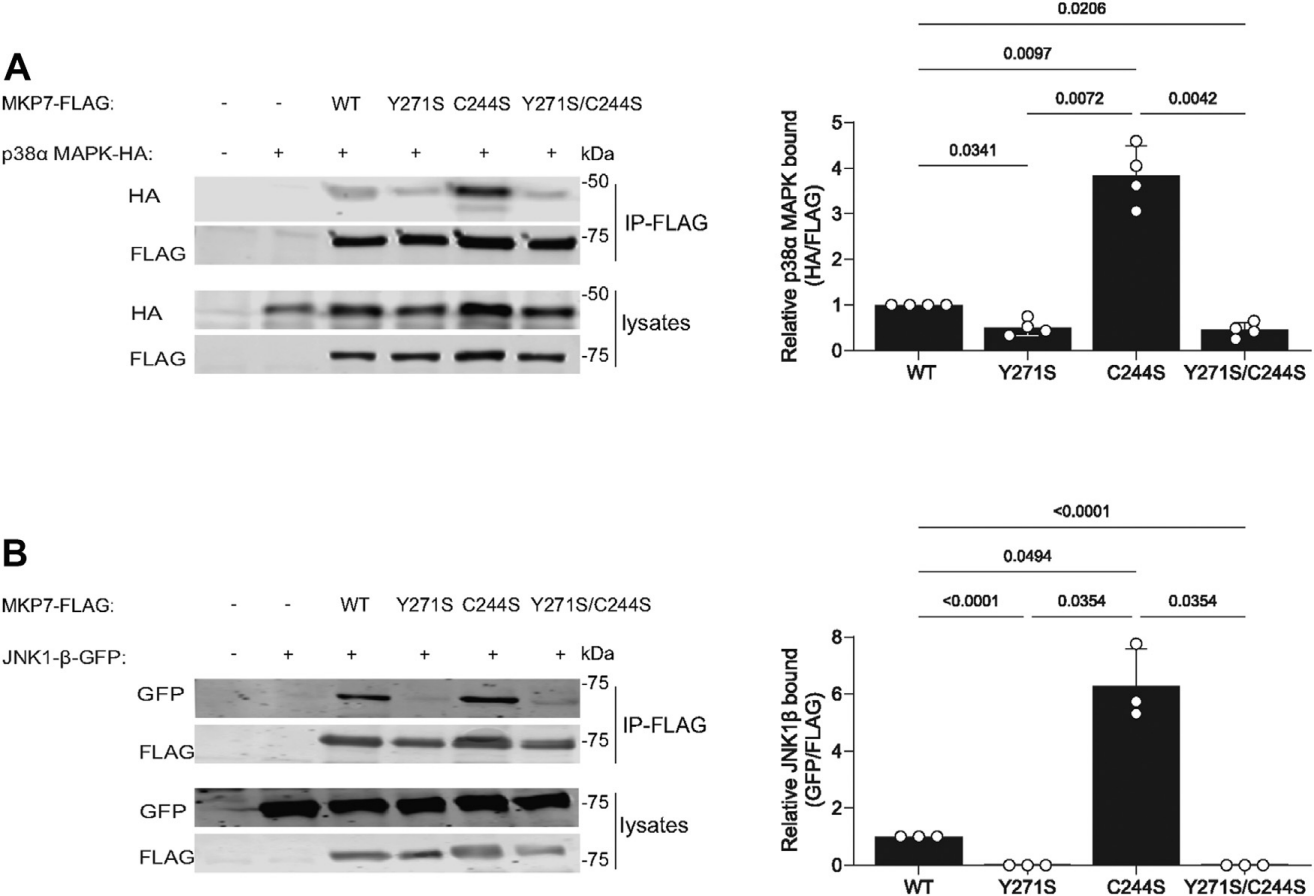
**Role of MKP7 Y271 on MKP7-MAPK binding at the catalytic cysteine residue.** Full-length FLAG-tagged MKP7 WT, Y271S, C244S, and the double Y271S/C244S mutant were transiently transfected with p38α MAPK-HA or JNK1-β-GFP into COS-7 cells for 48 h. Cells were harvested and lysed, followed by immunoprecipitation with anti-FLAG antibodies. *A*, anti-FLAG immune complexes and whole cell lysates were immunoblotted with anti-HA and anti-FLAG antibodies. *B*, anti-FLAG immune complexes and whole cell lysates were immunoblotted with anti-GFP and anti-FLAG antibodies. Graphs shown to the right represent quantitation of *A*, p38α MAPK-HA/FLAG-MKP7 and *B*, JNK1-GFP/FLAG-MKP7. Data represents the mean ± SEM of three to four independent experiments. Statistical significance shown was generated using a two-way ANOVA. MAPK, mitogen-activated protein kinase; MKP, MAPK phosphatase.

Our previous structural studies with MKP5 and the allosteric inhibitor showed a collapsed catalytic pocket by ∼18%, which prevented the dephosphorylation of the bound MAPKs presumably by disrupting the proper orientation of the catalytic cysteine with the incoming MAPK phospho-tyrosyl and phospho-threonyl residues (19). This model implies the MAPK interactions with MKP5 Y435 precede the dephosphorylation of the phospho-residues. We hypothesize that disruption of MAPK binding through Y271 mutation would mitigate the trapping effects of the catalytic cysteine. To investigate this potential mechanism of action on MKP7, we constructed double mutants with Y271S and C244S (YS/CS) to compare the interactions of MKP7 WT, Y271S, C244S, and YS/CS with p38α MAPK and JNK1. Following cotransfection of FLAG-tagged MKP7 variants with either p38α MAPK-HA or JNK1-GFP, we performed immunoprecipitation experiments followed by immunoblotting to detect for the presence of either p38α MAPK or JNK1. Mutation of Y271 in context of the C244S mutant resulted in the elimination of this mutant’s ability to effectively bind p38α MAPK and essentially reverts to the binding efficiency comparable to that of the Y271S mutant (Fig. 5*A*). Similarly, the YS/CS mutant completely abrogated the binding capacity of the C244S mutation to trap JNK1, resulting in undetectable levels of JNK1 in MKP7 immunoprecipitants as compared with the Y271S mutant (Fig. 5*B*). Taken together, these data suggest that Y271, and the putative allosteric pocket, is a major determinant for both MKP7 catalytic activity and binding interactions for the MAPKs.

### Effect of Y271 on MKP7/MAPK localization

**Figure 6:**
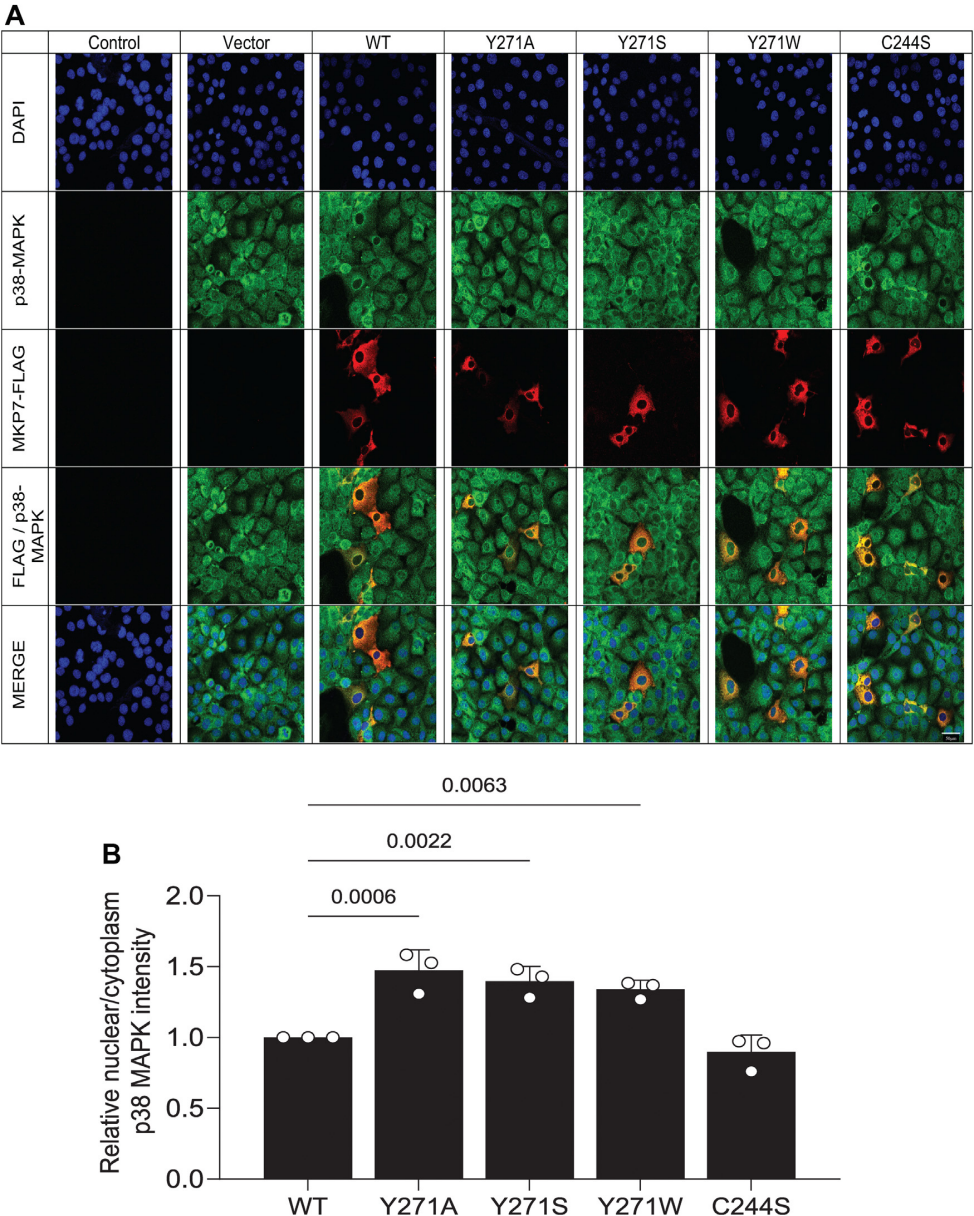

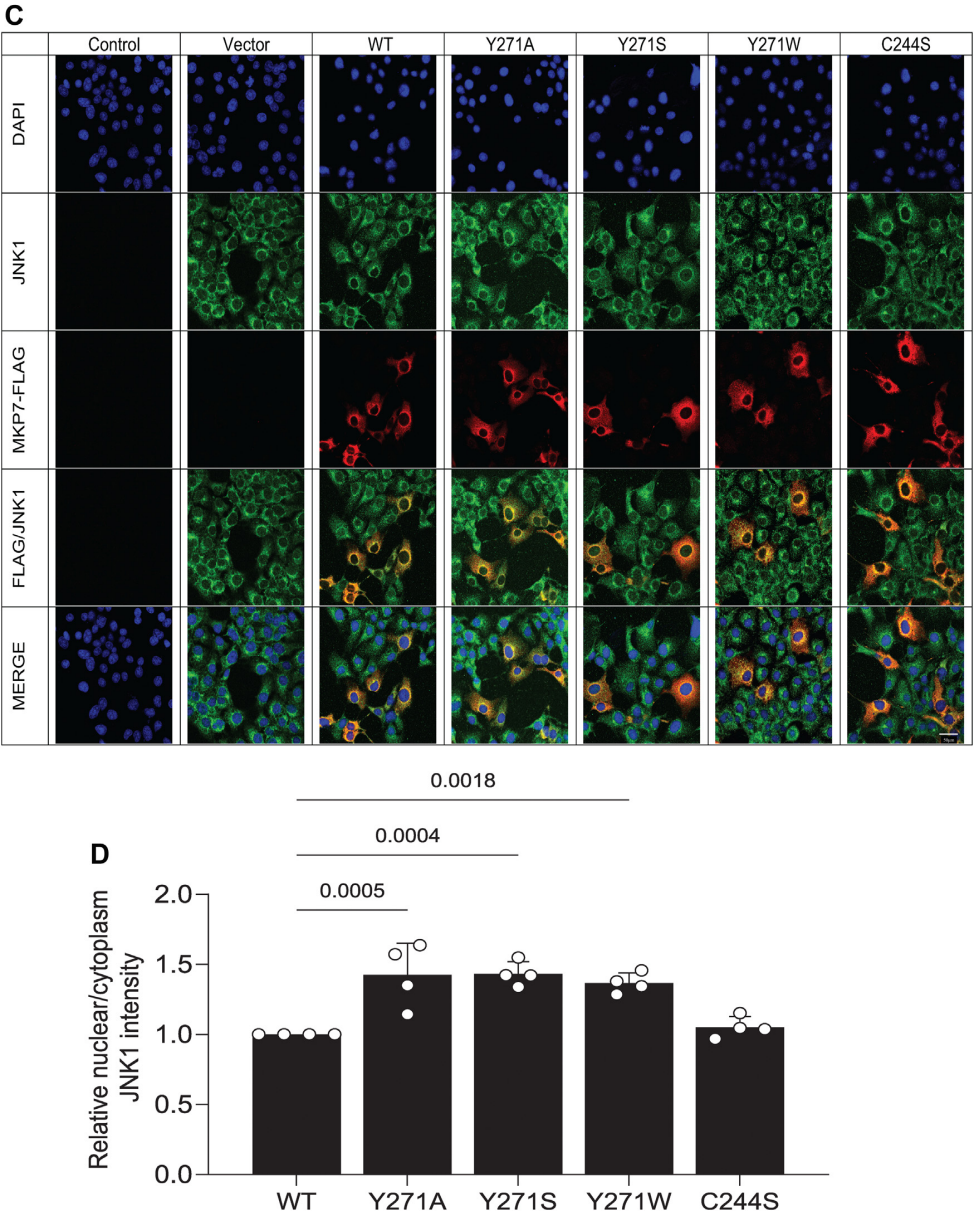
**Effect of MKP7 Y271 on MAPK localization.** Full-length FLAG-tagged MKP7 WT, Y271 mutants, and the C244S mutant were transiently transfected into COS-7 cells. Cells were fixed and stained with anti-FLAG antibodies (*red*) and DAPI (*blue*) for the detection of nuclei; *A* and *B*, anti-p38 MAPK or *C* and *D*, anti-JNK1/2 antibodies (*green*). The graphs shown represent the quantitation of nuclear/cytoplasmic ratio for (*A* and *B*) p38 MAPK and (*C* and *D*) JNK1/2 in FLAG-positive cells using CellProfiler. Data represent mean ± SEM from three independent experiments. Statistical significance was generated using one-way ANOVA. Image scale bar represents 400× magnification. MAPK, mitogen-activated protein kinase; MKP, MAPK phosphatase.

We next investigated whether Y271 in MKP7 exerts additional properties on the behavior of MKP7 beyond catalytic regulation and MAPK binding. First, we tested whether MKP7 Y271 mutants affected the subcellular localization of MKP7. The subcellular localization of MKP7 has been reported to be predominately cytoplasmic (20, 21). Consistent with these previous reports, we found that expression of MKP7 was cytoplasmic, however, the expression of the Y271 mutant of MKP7 did not affect its subcellular localization (Fig. 6, *A* and *C*). Next, we assessed whether Y271 influenced the distribution of the MAPKs between the cytoplasm and nucleus given our earlier observations that this residue was involved in MAPK binding (Fig. 4). It is conceivable that impaired MAPK/MKP7 binding due to Y271 mutation results in reduced cytoplasmic MAPK accumulation (20). To test this, FLAG-tagged MKP7 variants were overexpressed in COS-7 cells followed by indirect immunofluorescence staining for the expression of FLAG-tagged MKP7 and endogenous p38 MAPK or JNK. These experiments revealed that Y271 mutants failed to sequester either p38 MAPK or JNK in the cytoplasm and instead these MAPKs exhibited increased nuclear accumulation as compared with the distribution of these MAPKs in WT-expressing cells (Fig. 6, *A*–*D*). The observed redistribution of p38 MAPK and JNK to the nucleus in cells expressing the MKP7 Y271 mutants supports the notion that this residue plays a critical role in MAPK binding and further participates in the compartmentalization of localized MAPK dephosphorylation.

### Effects of MKP7 allosteric pocket mutants on MAPK signaling

**Figure 7:**
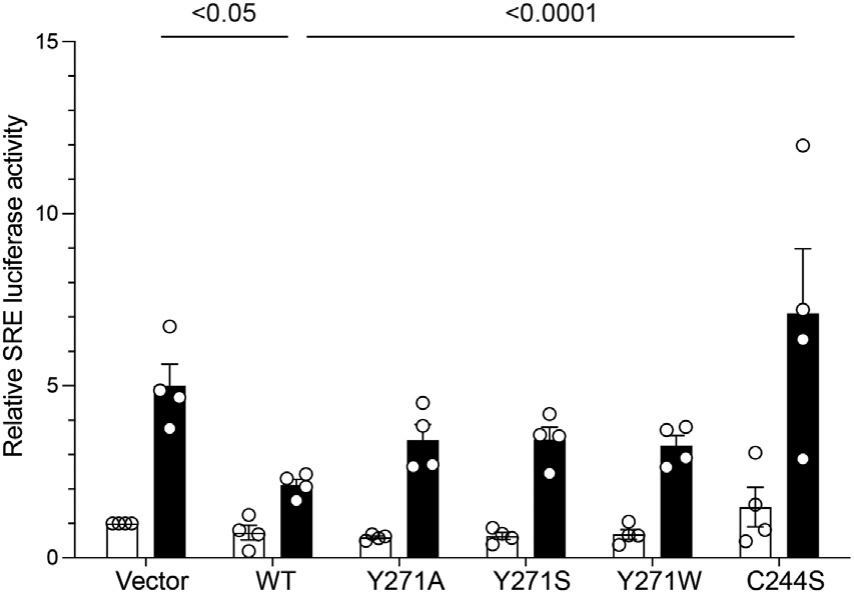
**Effect of MKP7 Y271 on serum-response element activation.** Full-length FLAG-tagged MKP7 WT, Y271 mutants and the C244S mutant were transiently transfected into 293 cells along with the SRE-luciferase reporter system. Cells were either left unstimulated (*open bars*) or stimulated with 10% serum (*closed bars*) for 4 h and luciferase activity measured and normalized to *Renilla*. Data shown are representative of four independent experiments and statistical significance was generated using a two-way ANOVA. MKP, MAPK phosphatase.

To determine the contribution of Y271 on MKP7-mediated MAPK signaling, we utilized a serum-responsive element (SRE) that responds to JNK, p38 MAPK, and ERK activity (27). The SRE drives transcriptional activation upon direct binding of the serum-response factor and the Ets-domain transcription factor Elk-1, both of which are activated upon phosphorylation by the MAPKs in response to diverse stimuli (27). 293 cells were transiently transfected with an SRE-luciferase along with either MKP7 WT, Y271A, Y271S, Y271W, or the catalytically inactive C244S mutant. Cells were rendered quiescent by serum deprivation and restimulated with 10% serum. In response to serum, SRE-mediated luciferase activity was induced by up to 5-fold in vector alone transfected cells, and MKP7 WT expressing cells significantly reduced this activation consistent with MAPK dephosphorylation (Fig. 7). Cells overexpressing the catalytically inactive MKP7 C244S mutant exhibited significantly enhanced SRE luciferase activity following serum stimulation as compared with MKP7 WT–expressing cells (Fig. 7). When the MKP7 allosteric site mutants Y271A, Y271S, and Y271W were overexpressed, they potentiated SRE luciferase activity following serum stimulation relative to that of MKP7 WT overexpressing cells albeit to a lesser extent than MKP7 C244S (Fig. 7). This observation that the allosteric pocket mutants potentiate SRE-mediated activity supports the interpretation that Y271 contributes to MAPK-mediated dephosphorylation and downstream signaling. Y271 was observed to not be as potent at enhancing serum-induced SRE activity as compared with C244S is likely due to these mutants being impaired in their ability to bind and sequester MAPK substrates (Figure 4, Figure 5, Figure 6). Collectively, these results demonstrate in a cellular context that the allosteric site defined by Y271 contributes to MKP7 catalysis and subsequently the inactivation of downstream MAPK signaling.

## Discussion

The MKPs are critical regulators of the MAPKs and members of this family have been suggested to be targets for human diseases such as cancer, cardiovascular and immunological diseases, and metabolic-related disorders. The identification of an allosteric site on MKP5 raised the possibility that other MKP family members may exhibit similar features. This supposition is bolstered by the observation that the primary amino acid sequence identified to constitute the allosteric site on MKP5 is highly conserved amongst all the active MKP family members. Most notably, Y435 which resides within the MKP5 allosteric site and is required for catalysis is conserved in 6 of the 10 catalytically active MKPs, with the other four members containing a conserved hydrophobic phenylalanine residue instead. To test whether this region of MKP5 that forms the allosteric pocket extends functionally to other MKPs, we tested the analogous position in MKP7, which has the highest similarity within the catalytic domain to MKP5. We found that mutation of MKP7 Y271 to either an alanine or serine dramatically reduced MKP7 catalytic activity either against *p*NPP or the p38α MAPK phospho-mimetic peptide by approximately 50%, whereas mutation to tryptophan reduced activity relative to WT by approximately 20%. These results suggested that maintaining the aromaticity at this site is a key factor for maintaining MKP7 activity. In agreement with the *in vitro* MKP7 activity data, we showed that overexpression of Y271 mutants of MKP7 in cells resulted in the hyperactivation of p38 MAPK and JNK1/2 but not ERK1/2 consistent with the substrate preference of MKP7 (20, 21) and potentiation of downstream activity of the SRE transcriptional element. Binding data on mutants targeting MKP7 Y271 indicates this residue plays an unanticipated but significant role in the binding interactions of p38α MAPK and JNK. The double mutant YS/CS, one at the allosteric residue and the other at the catalytic site, reveals information on the sequence of events that occurs, with the Y271 interaction to p38α MAPK and JNK preceding engagement of the phosphorylated MAPKs to the MKP7 catalytic site. Collectively, these results identify the analogous allosteric region of MKP5 that plays an essential role in its catalysis also exists within MKP7 to control catalysis and demonstrates that this region contributes to MAPK interactions.

We have demonstrated in MKP5 that the apo-structure of the PTP domain compared to the cocrystal structure of MKP5-PTP bound to the allosteric inhibitor decreases the catalytic pocket volume by ∼18% (19). The collapse of the catalytic pocket in MKP5 upon compound binding is due to a reorganization of residues in the active site associated with changes in loops α4–α5 and α5–β3 and Y435. Although we have not yet determined the crystal structure of MKP7 when bound to an allosteric inhibitor at this site, an analogous mutation at Y271 reveals a similar requirement for this residue for catalysis. Indeed, hydrophobicity in this “pocket” appears important given the less severe effects on catalysis incurred by the tryptophan mutant at this site. Thus, the analogous fold established in MKP7 confers a conserved region essential for MKP7 activity. Given the similarities in the primary sequence between MKP5 and MKP7 in this region, it is important to note that there are also differences that likely will be revealed when both proteins are compared in bound and unbound states. That such structural differences exist is intimated by our previous observation that when the MKP5 allosteric inhibitor was tested for activity against MKP7, it exhibited markedly reduced inhibitory efficacy (19). Thus, the putative allosteric pocket in MKP7 presumably loses critical interactions with the inhibitor that weaken potency. Inspection of the sequence surrounding Y271 indicates divergence that could account for this (Fig. 1). It will be important to fully understand the molecular basis for how MKP7 differs in this pocket of the phosphatase domain to that of MKP5 as it will inform the future development of specific MKP7 allosteric inhibitors.

The specificity of the MKPs for the MAPKs is largely driven by its ability to interact directly with MAPK *via* the KIM. Further, the selectivity of which MAPK is preferentially dephosphorylated is dictated, at least in part, by the affinity between the MKP–MAPK interactions. In the case of MKP7, it binds to both p38 MAPK and JNK and catalyzes dephosphorylation of these MAPKs. MKP7 has been shown to bind JNK *via* an F-X-F motif with the F285 playing the predominant role in JNK binding and F287 contributing to MKP7 activity with a lesser effect on JNK binding (23). In cellular immunoprecipitation assays, we found Y271 is also involved in MKP7-JNK and MKP7-p38α MAPK binding. Remarkably, a Y271 mutation completely abolished JNK binding, suggesting that this site plays a critical role, but not necessarily to the exclusion of the contribution of F285/287, for JNK binding. Similar results were also seen for p38 MAPK binding in cells but in this context, there was reduced dependency for Y271. Interestingly, the effect of the Y271 mutants on MAPK binding is qualitatively comparable to that of mutations within the DII MAPK docking domain (LXL motif) of MKP7, which leads to a dramatic decrease in both p38 MAPK and JNK binding to MKP7 (22). Collectively, these results support a major contribution of the Y271 region as a MAPK docking site. Given that MKP7 binds to both p38 MAPK and JNK through Y271 we investigated whether this binding serves to influence the subcellular localization of MKP7. We found that mutation of Y271 did not directly affect the subcellular localization of MKP7. However, all three Y271 mutants resulted in an increase in the nuclear/cytoplasmic ratios of both p38 MAPK and JNK, indicating that loss of MKP7 binding facilitated nuclear accumulation of these MAPKs. MKPs such as MKP3 and HVH5 have been shown to anchor the ERK proteins in the cytoplasm and nucleus respectively, through the N-terminal KIM, indicating that there is precedence for MKPs influencing MAPK localization (28, 29). These data reinforce the functional effects of Y271 as a MAPK binding/interaction domain that facilitates MAPK dephosphorylation and MAPK subcellular localization.

For most of the MKPs, after binding/substrate recognition through docking motifs on the MAPKs, the catalytic cysteine attacking the substrate’s phosphate group leads to conformational changes within the catalytic pocket to accommodate the substrate (16, 24, 30). For example, MKP3 undergoes a profound induced-fit to accommodate and appropriately orient the phosphorylated activation loop of ERK1/2, and other MKPs display similar behavior (30). To explore whether Y271 impacts substrate access to the active site pocket, we overexpressed the double mutant YS/CS to determine the influence of MAPK binding at this site on engagement to the catalytic cysteine residue. Remarkably, we discovered that in context of the Y271 mutant, the MAPK-trapping ability of the MKP7 CS mutant was completely abrogated. This suggests that Y271 is essential for MAPK binding and that binding at this site is a prerequisite event to engagement of the catalytic cysteine residue by phosphorylated p38 MAPK and JNK.

In addition to previously described docking domains on MKP7 (20, 21, 22), this new region (19) identifies a site that is critical for proper substrate recognition and binding that precedes substrate access to the active site. This pocket neighbors the FXF motif on MKP7 that is thought to be a substrate recognition site for JNK and p38 MAPK as a well as a site required for alignment of the MKP7 active site (23). The Y271 mutant appears unique in that while it does effectively eliminate MKP7-JNK binding, it also reduces p38 MAPK binding (JNK>>p38). It is likely that this site mediates MAPK binding prior to catalysis with a distinct modality. Across the MKPs, MAPK binding has been assigned to the N- and C-terminus D-motifs outside the PTP domain (22) and to the FXF motif within the PTP domain (23, 31). The assessment of this site as a region affecting MKP5 catalysis and a potential regulatory region on other MKPs (19) is supported by our characterization of this analogous site in MKP7. Consistent with the role of Y271 in regulating MKP7 activity, overexpression of Y271 resulted in a dominant-negative effect on SRE-mediated transcriptional activity. Interestingly, the effect of Y271 on SRE transcriptional activity in response to serum stimulation was lower than that achieved by the C244S mutant, which is both substrate-trapping and catalytically dead. Thus, it is reasonable to suggest that the enhancement of Y271 mutants on SRE activity is absent that of the substrate-trapping properties that exist for C244S. Indeed, one could assert that the Y271 mutants’ effect on SRE-mediated transcriptional activity was a closer reflection of solely impaired MKP7 activity as these mutants are not confounded by the substrate-trapping properties of C244S. Much work using the catalytically inactive mutant of the MKPs has been performed and conclusions derived from such studies with little interpretation to account for the substrate-trapping issues. Given these issues, it would be of interest to test similar MKP7 allosteric site mutants in other MKPs for their effects on MAPK-mediated downstream signaling which should solely reflect the contribution of enzymatic activity.

MKP7 has been mapped to *12p12* (20, 21), a tumor suppressor locus, whose deletion has been associated with acute lymphoblastic leukemia as well as several hematological malignancies including lung, breast, and ovarian carcinomas (32). MKP7 has been found to be upregulated in several cancers and has been shown to increase the resistance of cancer cells to senescence (33, 34) and as such is an attractive anti-cancer therapeutic target. While there have been attempts to develop active site MKP7 inhibitors (35), these inhibitors have only achieved low micromolar potency and weak specificity when tested against other DUSP family members. Exploration of the allosteric region in MKP5 has led to the development of an inhibitor that has specificity for MKP5 with an IC50 that is >100-fold more potent than MKP7 due to key amino acid residue differences within this pocket (19). This suggests the conserved MKP allosteric pocket can be exploited to develop effective inhibitors with high selectively for MKP7. Future work to solve the *apo*-crystal structure of both WT MKP7 and the Y271 mutants, in addition to mutational analysis of neighboring residues, could lead to structural insights into how Y271 facilitates MKP7 catalysis. This information will prove to be essential for a broader understanding of the regulatory mechanisms of the MKPs.

## Experimental procedures

### Expression vectors and cloning

pLX302-MKP7-V5 puro was a gift from Kevin Janes (Addgene plasmid # 87771; RRID:Addgene_87771) (36) and used to clone MKP7 PTP domain (156–301 aa) into pet28a, using the primers: 5′-CGCGGATCCATGAACATTGGGCCAACCCGAATTC and 3′- CCGCTCGAGTCAAGTCTGGTTCTTAATCTTCTTCTCA. Y271 PTP mutants were generated using QuickChange II (Agilent) site-directed mutagenesis using the following primers:

Y271A primers: 5′-TTAGATGAAGCTGCAAGATTTGTGAAAGA.

3′TCTTTCACAAATCTTGCAGCTTCATCTAA.

Y271S primers: 5′TTAGATGAAGCTTCTAGATTTGTGAAAGAA.

3′TTCTTTCACAAATCTAGAAGCTTCATCTAA.

Y271W primers: 5′TTAGATGAAGCTTGGAGATTTGTGAAAGA.

3′TCTTTCACAAATCTCCAAGCTTCATCTAA.

Full-length FLAG-tagged MKP7 WT (1–665 aa) in pCDNA3.1 was ordered from Genscript (Clone ID OHu20957) and was used for mutagenesis to generate Y271A, Y271S, and Y271W using the same primers for MKP7 PTP mutants with QuickChange II (Agilent) site-directed mutagenesis protocol and transformed into XL-GOLD Ultracompetent cells (Agilent cat#200317). FLAG-MKP7 C244S mutant was generated using primers: 5′ATGTGTTCTAGTGCACTCTTTAGCTGG and 3′ CCAGCTAAAGAGTGCACTAGAACACAT. Double mutant, Y21S/C244S was generated using FLAG-tagged MKP7 C244S as a template using QuickChange II (Agilent) site-directed mutagenesis protocol and the use of the same Y271S primers used to generate the single mutant. HA-tagged p38α MAPK (1–360 aa) was cloned into pcDNA3.1, and HA-tagged ERK2 (1–360 aa) was cloned into pCG5 for mammalian cell expression. GFP-tagged JNK1 in pEGFP-C2 vector was a gift from Rony Seger (Addgene plasmid # 86830). The SRE luciferase reporter vector (5XSRE-luciferase) was used as described previously (37) and the pRL-*Renilla* vector for purposes of normalization was purchased from Promega.

### Protein expression and purification

pet28a vector, His-MKP7 PTP-WT, Y271A, Y271S, and Y271W were transformed into BL21 GOLD DE3 (Agilent cat#230132). Cells were grown in LB with kanamycin (50 μg/ml) at 37 °C. For expression of WT, 800 μM IPTG was added, while for Y271 mutants, 400 μM IPTG was added to induce protein expression and cells were harvested after overnight incubation at 16 °C. Cells were lysed and purified by His-tag affinity purification in a gravity flow column using TALON Metal Affinity Resin (Takara cat#635502) as previously described (19).

### Protein folding assessment (circular dichroism)

Evaluation of protein folding was carried out using circular dichroism. MKP7-PTP constructs were diluted to a final volume of 400 μl in storage buffer (20 mM tris (pH 7.4), 150 mM NaCl, 5% glycerol, and 5 mM DTT) and were analyzed for circular dichroism absorption by an Applied Photophysics Chirascan circular dichroism spectrophotometer (Model 215, AVIV Instruments Inc). Curves for each construct were normalized so that the minimum ellipticity is equal.

### Cell culture

Cell-based assays utilized COS-7 or 293 cells grown at 37 °C at 5% CO_2_. Cells were maintained in Dulbecco’s modified eagle’s medium (DMEM) (cat#11965092) supplemented with 10% fetal bovine serum (FBS), 1% sodium pyruvate (Gibco cat#11360070), and 1% penicillin-streptomycin (Gibco cat#15140122).

### Phosphatase activity assay

For malachite green assays, a phospho-p38α MAPK peptide (Asp-Asp-Glu-Met-pThr-Gly-pTyr-Val-Ala-Thr-Arg) was used. Experiments were performed in a 384-well plate in 10 μl solution. Five microliters of MKP7 PTP at 2.5 μM were added to wells with 5 μl of pTpY peptide (p38α MAPK phospho-mimetic) at 100 μM for 30 min at 30 °C. After incubation, 40 μl of malachite green was added to all wells and incubated at room temperature for 10 min followed by absorbance measurement at 620 nM. Proteins were diluted in a PTP buffer composed of 50 mM Tris pH 7.2, 1 mM EDTA, 0.1% 2-beta-mercaptoethanoll, 0.01% TritonX-100. Malachite green solution was composed of 0.045% Malachite green (Sigma #M-9636) in H_2_O and filtered through a 0.2 μM unit. Ammonium Molybdate solution consisted of 4.2% Ammonium Molybdate (Sigma A-7302) in 4 M HCl per 100 ml. A 2.5× dilution of the Ammonium Molybdate solution into the malachite green solution created the working assay Malachite green solution. For *p*NPP assays, experiments were performed in 384-well plates in 25 μl. Five microliters of MKP7 PTP at 10 μM were added to wells with 20 μL of *p*NPP solution comprised of 10 mM *p*NPP, 24 mM Hepes (pH 7.4), 120 mM NaCl, and 5 mM DTT. The reaction mix was incubated at 37 °C for 30 min and was quenched with 75 μl NaOH at a concentration of 0.2 M. The absorbance at 405 nM was taken immediately after terminating the reaction.

### Immunoblotting and immunoprecipitation

COS-7 cells were seeded at a density of 1 × 10^6^ in 100-mm dishes and maintained in DMEM supplemented with 10% FBS for 24 h prior to transfection. Cells were transfected with 8.5 μg DNA of either pcDNA3.1 or cFLAG-MKP7 (WT or mutants) using FuGENE transection reagent as per manufacturer’s instructions. Twenty four hours after transfection, cells were washed once with PBS and starved overnight in 0.1% FBS in DMEM. Next day, cells were washed with PBS followed by incubation with 500 mM sorbitol in 0.1% FBS in DMEM for 30 min before lysis followed by immunoblot analysis for detection of endogenous p38 MAPK, JNK1/2, and ERK1/2. COS-7 cells were seeded at a density of 8.5 × 10^5^ in 100-mm dishes and maintained in DMEM supplemented with 10% FBS for 24 h before transfection. Cells were cotransfected with 6.8 μg DNA of either pcDNA3.1 vector or cFLAG-MKP7 (WT or mutants) expression vector and 3.4 μg of either pcDNA3-HA-p38α MAPK, pEGFP-JNK1β, or pCG-HA-ERK2 using FuGENE transection reagent as per manufacturer’s instructions. Cells were transfected for 48 h in 10% FBS DMEM before harvest. Cells were lysed on ice using Pierce IP lysis buffer (cat# 87787). Cell lysates were precleared with protein-G Sepharose (Sigma cat# GE-17-0618-01) followed by overnight incubation with anti-FLAG M2 monoclonal antibody (Sigma cat# F3165). Lysates were then incubated with protein-G beads for 1 h followed by boiling of samples in 1× sample buffer for immunoblotting detection of p38α MAPK-HA or ERK2-HA using anti-HA (12CA5) monoclonal antibody (Roche cat#11583816001), JNK-GFP using anti-GFP monoclonal antibody (Cell Signaling Technologies # 2955), and anti-FLAG polyclonal antibody (Sigma cat#7425).

### Immunofluorescence assay

COS-7 cells were seeded at a density of 1 × 10^5^ on coverslips in 12-well dishes maintained in DMEM supplemented with 10% FBS for 24 h before transfection. Cells were transfected with 1.5 μg DNA of either pcDNA3.1 vector or cFLAG-MKP7 (WT or mutants) expression vector and FuGENE transfection reagent as per manufacturer’s instructions. Twenty four hours after transfection, cells were washed once with PBS and placed in 0.1% FBS in DMEM for starvation overnight. Next day, cells were washed with PBS followed by cell permeabilization and fixation using ice cold methanol/acetone (1:1). Cells were blocked for 1 h in 5% normal goat serum (Gibco cat#16210064) followed by overnight incubation with anti-FLAG M2 monoclonal antibody (Sigma cat#F3165) (1:500) and anti-p38 MAPK (Cell Signaling Technologies cat#8690) (1:50) or anti-FLAG polyclonal antibody (Sigma cat#7425) (1:500) and anti-JNK1 monoclonal antibody (Santa Cruz biotechnology cat#398989) (1:50). Cells were fixed on slides using VECTORSHIELD mounting medium with DAPI (cat#H-1500-10) to stain for nuclei.

### Immunoblotting analysis

For Western blot analysis, cells were lysed on ice in lysis buffer containing 25 mM Tris–HCl (pH 7.4), 150 mM NaCl, 1 mM EDTA, 5% glycerol, 0.5% Tween-20, 1 mM Na_3_VO_4_, 10 mM NaF, 1 mM PMSF, 1 mM benzamidine, 1 μg/ml pepstatin A, 5 μg/ml leupeptin, and 5 μg/ml aprotinin. Cell lysates were clarified by centrifugation at 20,000*g* at 4 °C for 15 min. Protein concentration was determined using Pierce BCA kit. Protein samples were boiled in 1× sample buffer and separated using SDS-PAGE and transferred to nitrocellulose membranes by semi-dry transfer for 1 h. Membranes were incubated at room temperature in 1% Casein in TBS (BioRad cat #1610782) blocking buffer followed by overnight incubation with primary antibodies (1:1000 in 5% BSA in TBST). Proteins were visualized after incubation with fluorescence secondary antibodies and image processing using LI-COR image Studio Software. Primary antibodies used from Cell Signaling were p38 MAPK (Thr180/Tyr182) (3D7) (cat# 9215), pERK1/2 (cat#9101), ERK1/2 (cat#9107), pSAPK/JNK (cat#4668), JNK1 (cat#3708), GFP (cat#2955) and from Sigma flag M2 (cat#F3165) and M2 (cat#F7425), and Roche: HA 12CA5 (cat#11583816001).

### CellProfiler immunofluorescence images analyses

Analysis of immunofluorescence images of COS-7 cells stained for MKP7 (FLAG), p38 MAPK or JNK1/2, and DAPI (cell nuclei) required the single channel of each image to be uploaded to the program to quantify pixel/color intensity. The CellProfiler Program used DAPI (blue) images to identify all cells/nuclei in each image frame and used the red channel images to identify all FLAG-MKP7–positive cells as well as individual cell boundaries. Thus, both DAPI and FLAG images allowed for individual cell nuclei and cytoplasm to be identified. The green channel intensity was measured and quantified in cells and the ratio of nuclear/cytoplasmic (green) intensity for either p38 MAPK or JNK were measured in cells.

### SRE luciferase reporter assay

SRE luciferase activity was measured using the Luciferase assay system kit from Promega as previously described (37). 293 cells were seeded in 12-well dishes and cotransfected using FuGENE with 5XSRE-luciferase (0.5 μg), pRL-*Renilla* (2 ng), and either pcDNA3.1 FLAG-MKP7 WT, Y271A, Y271S, Y271W, or C440S (1.0 μg). After 24 h, cells were serum-starved overnight followed by stimulation for 4 h with 10% FBS. Cells were lysed and luciferase activity measured according to the manufacturer’s instructions.

### Statistical analyses

No statistical methods were used to predetermine sample size. The number of samples (n) used in each experiment is shown. All *in vitro* experiments were performed at least three times independently. Statistical analysis and graphing were performed using GraphPad Prism 9.4.1 software. We did not estimate variations in the data. The variances are similar between the groups that are being statistically compared. All data represent the means ± SEM. For *p* value determinations, we used one-way or two-way ANOVA with multiple comparisons.

## Data availability

All data are included in this article. Further information and requests for resources and reagents should be directed to and will be fulfilled by Anton M. Bennett (anton.bennett@yale.edu).

## Conflict of interest

The authors declare that they have no competing interests with the contents of this article.
